# Systematic review and meta-analysis of the utility of long non-coding RNA GAS5 as a diagnostic and prognostic cancer biomarker

**DOI:** 10.18632/oncotarget.19040

**Published:** 2017-07-06

**Authors:** Wei Li, Na Li, Ke Shi, Qiong Chen

**Affiliations:** ^1^ Department of Geriatrics, Xiangya Hospital of Central South University, Changsha 410008, Hunan Province, China; ^2^ Department of Pathology, The First Affiliated Hospital of Hunan University of Medicine, Huaihua 418000, Hunan Province, China

**Keywords:** GAS5, cancer, clinical outcome, diagnosis, prognosis

## Abstract

The growth arrest-specific 5 transcript (GAS5) is a long non-coding RNA (lncRNA) involved in the control of cell cycle progression and apoptosis in a wide variety of cells. To determine the clinical value of GAS5 expression in cancer patients, we performed a systematic review and meta-analysis exploring its association with the diagnosis, prognosis, and clinicopathological characteristics of cancer. Ten articles on prognosis, 15 on clinicopathology, and 5 on diagnosis were analyzed. Overall results showed that decreased GAS5 expression associated with unfavorable overall survival (OS) (HR = 2.50, 95%CI: 1.85–3.38, *P* < 0.001) and disease-free survival (DFS) (HR = 2.24, 95%CI: 1.58–3.18, *P* < 0.001) in several tumor types. Down-regulation of GAS5 correlated with poor recurrence-free survival (RFS) in hepatocellular carcinoma (HR = 2.40, 95%CI: 1.27–4.54, *P* = 0.007), and was associated with lymph node metastasis (OR = 1.92, 95% CI: 1.44–2.57, *P* < 0.001), distant metastasis (OR = 2.7, 95% CI: 1.05–6.97, *P* = 0.040), poor clinical stage (OR = 0.26, 95% CI: 0.18–0.38, *P* < 0.001), larger tumor size (OR = 3.21, 95% CI: 2.08–4.95, *P* < 0.001), and poor tumor differentiation (OR = 1.98, 95% CI: 1.40–2.80, *P* < 0.001). Pooled results of diagnostic data analysis showed that GAS5 exhibited a sensitivity of 0.76 and specificity of 0.64 for cancer diagnosis, and an area under the curve of 0.76 (95% CI: 0.72–0.80) indicated moderate diagnostic accuracy. This meta-analysis suggests GAS5 lncRNA may be a useful diagnostic and prognostic cancer biomarker, and may be especially useful for identifying patients prone to developing lymph node or distant metastasis.

## INTRODUCTION

Growth arrest-specific transcript 5 (GAS5) is a widely expressed long non-coding RNA (lncRNA) transcript of about 630 nt in length, encoded by the *GAS5* gene located on chromosome 1q25. This lncRNA was initially isolated from a subtraction cDNA library of growth-arrested cells and identified as a potential new tumor inhibitor [1]. GAS5 stimulates apoptosis during serum starvation or growth factor deprivation by acting as a glucocorticoid response element (GRE) decoy, competing with GRE sequences in the DNA for binding to the glucocorticoid receptor (GR) [2]. Studies like the one by Mourtada-Maarabouni et al., showing that overexpression of lncRNA GAS5 induces apoptosis and slows the cell cycle in human T-cell lines [3] have reinforced the notion that lncRNA GAS5 plays a key role in cell growth arrest and apoptosis.

In recent years, lncRNA GAS5 has attracted considerable interest due to evidence indicating that its downregulation in a wide variety of neoplasms, including ovarian cancer [4, 5], prostate cancer [6, 7], non-small cell lung cancer [8, 9], gastric cancer [10, 11], colorectal cancer [12, 13], and hepatocellular carcinoma [14, 15], is generally associated with poor prognosis [10–14]. Several studies also demonstrated that upregulation of GAS5 expression inhibits tumor cell proliferation and promotes apoptosis [5–8, 12, 14–16]. By interacting with many biological molecules, including certain steroid hormone receptors and miRNAs, GAS5 modulates gene expression and impacts pivotal regulatory pathways of cell survival [17]. These discoveries are consistent with lncRNA GAS5 being a ubiquitous tumor suppressor and prognosis marker.

However, due to insufficient sampling and/or methodological limitations, published studies on the association of lncRNA GAS5 with cancer are often inaccurate and/or inadequate. To address this shortcoming, we systematically collected all relevant publications and conducted the present meta-analysis addressing the relation between GAS5 expression and cancer diagnosis, clinicopathological characteristics, and clinical outcomes. Our overall results confirm a correlation between reduced GAS5 expression and poor clinical outcomes, and reveal an association with increased likelihood of lymph node metastasis (LNM) and distant metastasis (DM) in patients with early stage tumors.

## RESULTS

### Study characteristics

**Figure 1:**
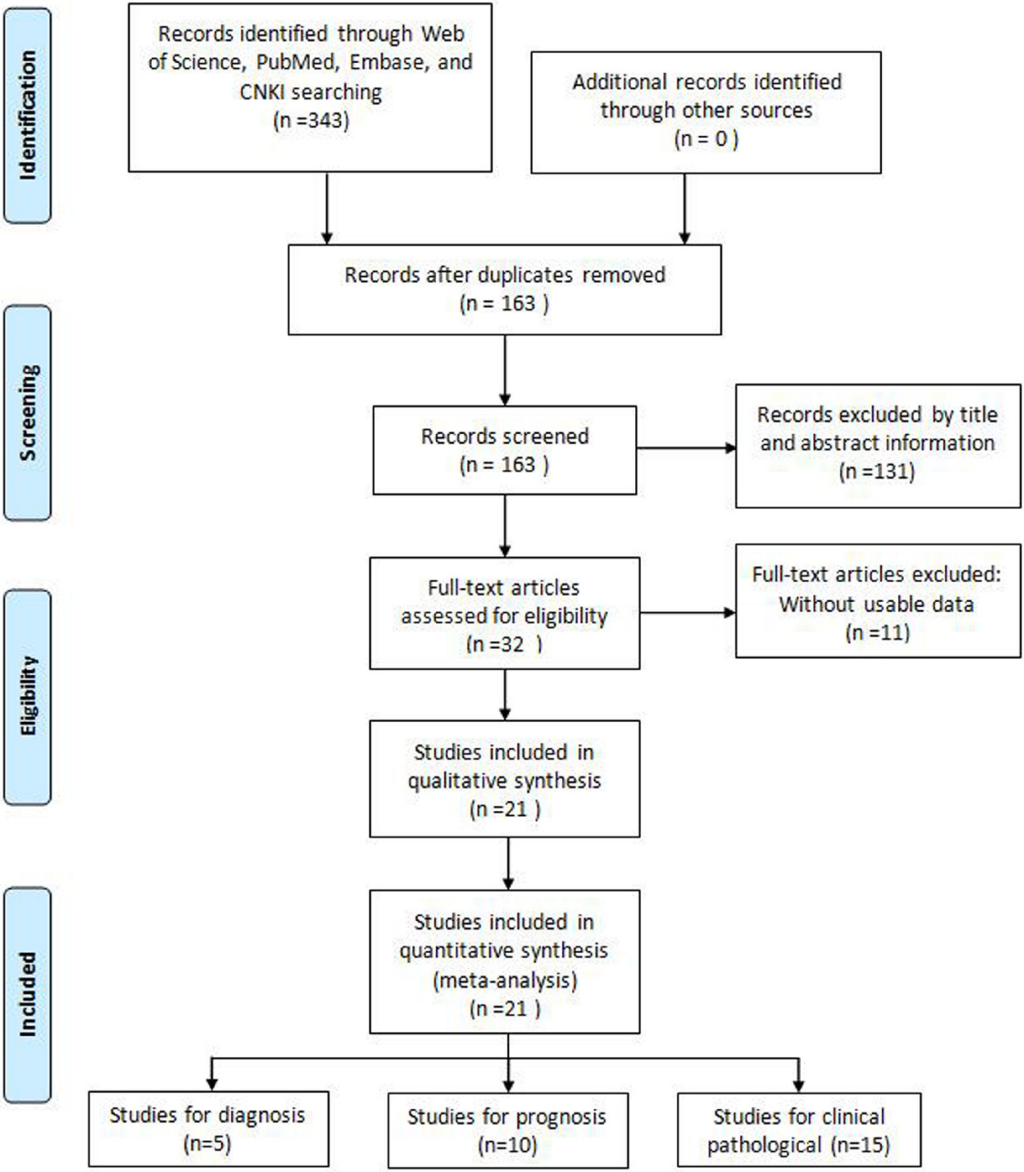
Flow diagram of the literature search and selection

A total of 343 potentially relevant articles were retrieved from Embase, PubMed, Web of Science, and China Knowledge Resource Integrated (CNKI) databases. After removing duplicates, 163 records were preserved. Following title and abstract revision, 131 records were excluded. Subsequently, from the 32 remaining studies 11 were excluded due to incomplete data. Finally, a total of 21 studies, including 10 on prognosis [4, 9–12, 14–16, 18, 19], 15 on clinicopathological features [4–6, 8, 10–12, 14, 15, 18–23], and 5 on diagnosis [13, 23–26], met the criteria used for inclusion in the meta-analysis (Figure 1). All 21 studies came from China, and addressed 8 different tumor types: hepatocellular carcinoma (HCC), non-small cell lung cancer (NSCLC), ovarian cancer (OC), colorectal cancer (CRC), gastric cancer (GC), cervical cancer (CC), bladder transitional cell carcinoma (BTCC), and multiple myeloma (MM). In all cases GAS5 expression was detected by qRT-PCR.

### Correlation of GAS5 expression with clinical outcome

**Figure 2:**
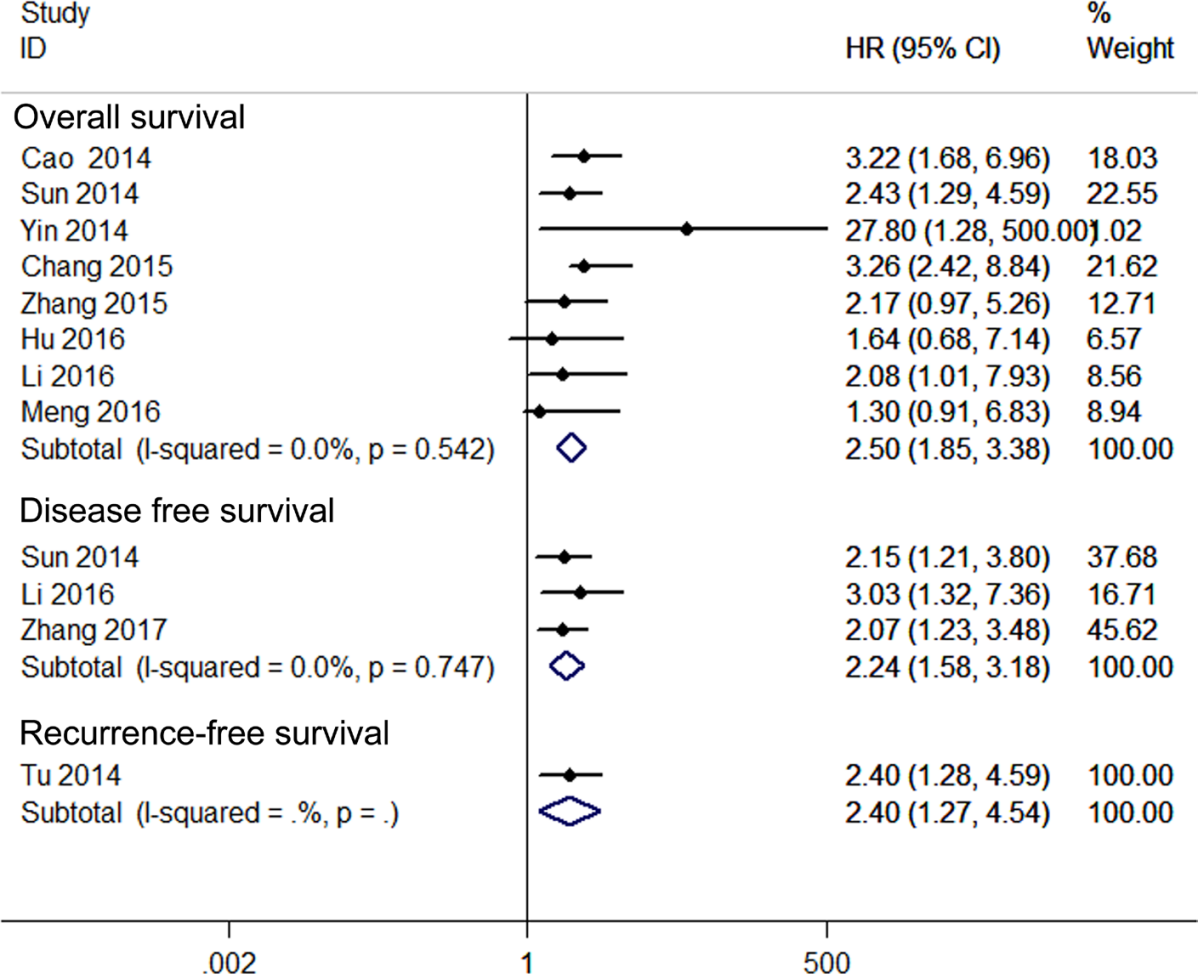
Forest plot for the relationships between decreased GAS5 expression and OS /DFS/RFS

**Figure 3:**
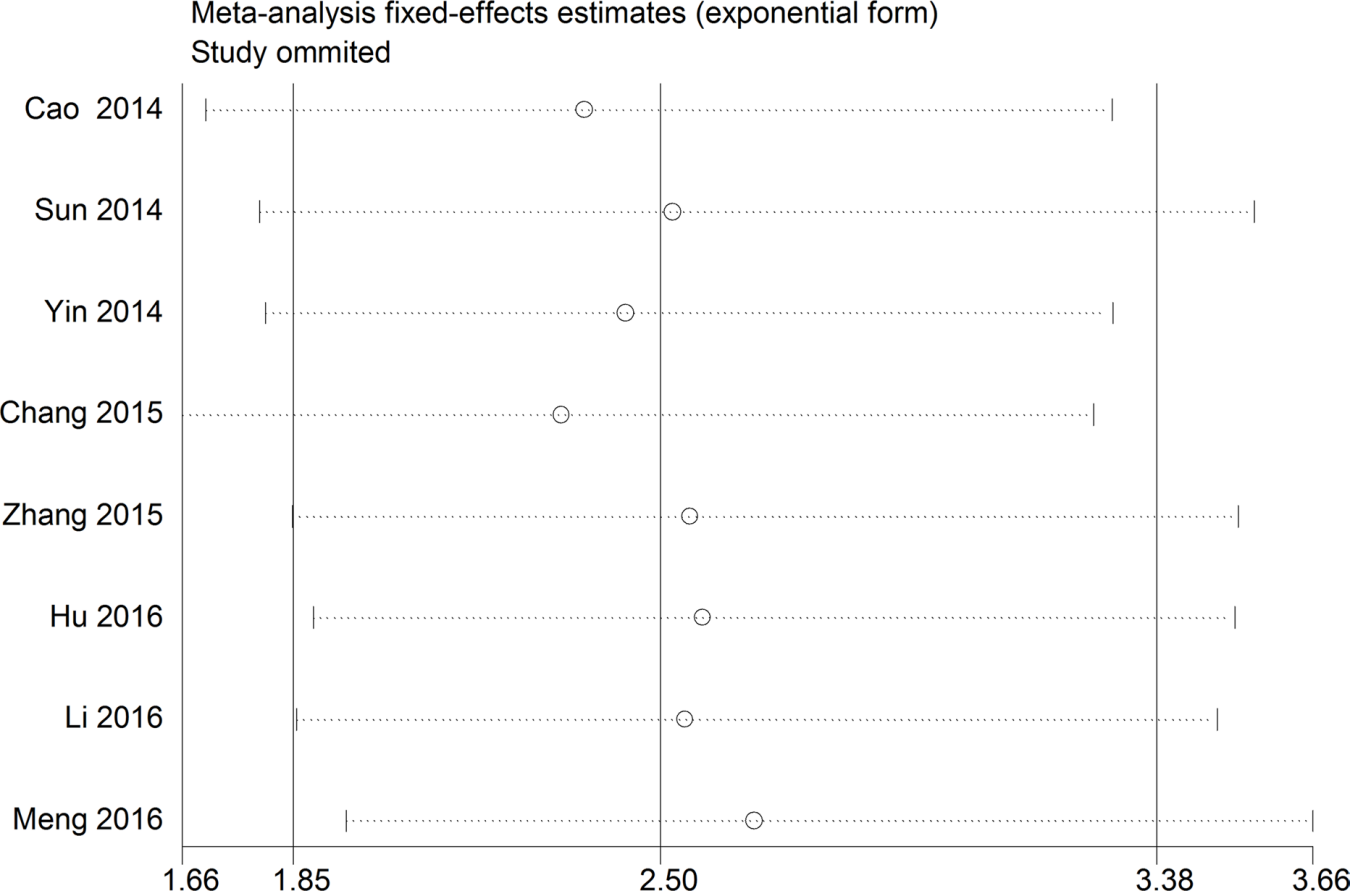
The sensitivity analysis for the meta-analysis of OS in tumor patients

**Figure 4:**
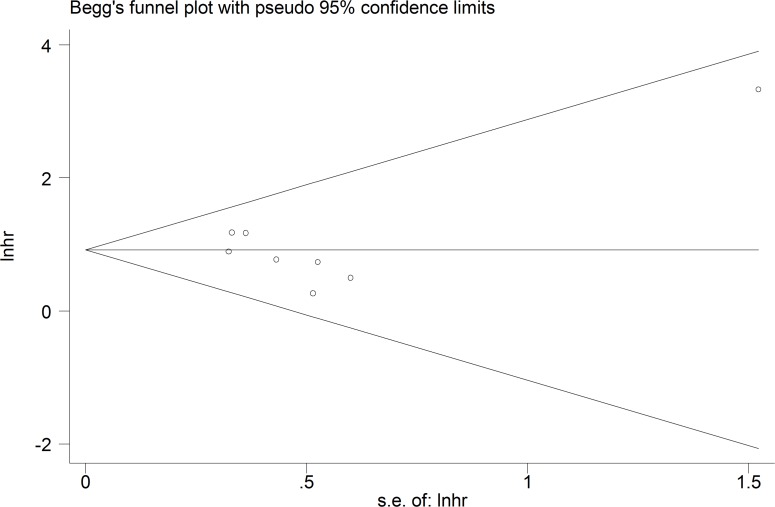
Funnel plot analysis of potential publication bias for meta-analysis of OS in tumor patients

**Figure 5:**
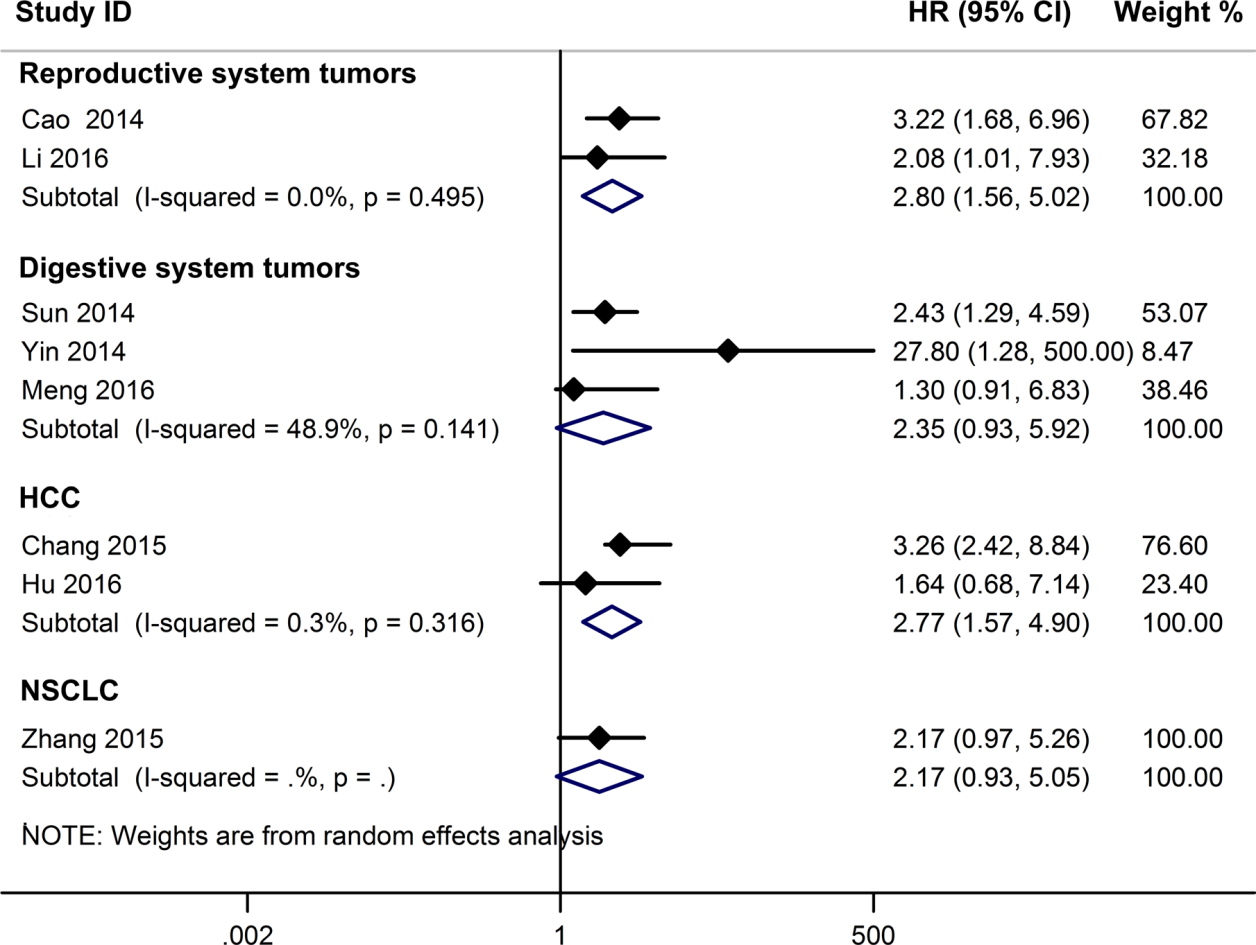
Subgroup analyses for OS according to cancer type

A relationship between GAS5 expression and overall survival (OS) was detected in 8 studies comprising 507 patients (Table 1). The pooled HR indicated a negative association between GAS5 expression and OS (HR = 2.50, 95% CI: 1.85–3.38, *P <* 0.001; fixed effects model) (Figure 2). A sensitivity analysis was carried out to evaluate the robustness of the merged results, which were not significantly affected by exclusion of any single study. This indicated that the pooled OS HR was robust (Figure 3). A Begg’s funnel plot test (Figure 4) further showed no obvious publication bias between the included studies (*Pr* > |z| = 0.536). We also performed subgroup analyses according to cancer type. As shown in Figure 5, compared with the pooled HR for all cancers, GAS5 displayed a stronger correlation with poor OS in the subgroups of reproductive system tumors (HR = 2.80, 95% CI: 1.56–5.02, *P <* 0.001), and in HCC (HR = 2.77, 95% CI: 1.57–4.90, *P <* 0.001).

**Table 1:** Main characteristics of the eligible prognosis studies

Study	Tumor type	Sample size	Test Method	Cut-off	Outcome measure	Analysis method	HR estimation	Follow-up (months)
Cao 2014	CC	102	qRT-PCR	median value	OS	Multivariate	Direct	∼60
Sun 2014	GC	89	qRT-PCR	median value	DFS, OS	Multivariate	Direct	∼40
Tu 2014	HCC	71	qRT-PCR	median value	RFS	Multivariate	Direct	∼60
Yin 2014	CRC	66	qRT-PCR	median value	OS	Multivariate	Direct	∼60
Chang 2015	HCC	50	qRT-PCR	median value	OS	Multivariate	Direct	∼60
Zhang 2015	NSCLC	50	qRT-PCR	median value	OS	Multivariate	Indirect	∼70
Hu 2016	HCC	32	qRT-PCR	median value	OS	Multivariate	Indirect	∼30
Li 2016	OC	63	qRT-PCR	median value	DFS, OS	Multivariate	Indirect	∼36
Meng 2016	GC	55	qRT-PCR	median value	OS	Multivariate	Indirect	∼ 36
Zhang 2017	BTCC	82	qRT-PCR	median value	DFS	Multivariate	Direct	∼ 60

CC: cervical cancer; GC: gastric cancer; HCC: hepatocellular carcinoma; CRC: colorectal cancer; NSCLC: non-small cell lung cancer; OC: ovarian cancer; BTCC: bladder transitional cell carcinomas; OS: overall survival; DFS: disease free survival; RFS: recurrence-free survival; HR: hazard ratio.

Three studies including 234 patients reported HRs for disease-free survival (DFS). The overall result revealed that decreased GAS5 expression could predict poor DFS (HR = 2.24, 95%CI: 1.58–3.18, *P <* 0.001) (Figure 2). Only one study, comprising 71 patients, reported HR for recurrence-free survival (RFS). In such report, down-regulation of GAS5 also correlated with poor RFS (HR = 2.40, 95% CI: 1.27–4.54, *P* = 0.007) (Figure 2).

Hepatocellular carcinoma; NSCLC: Non-small cell lung cancer; OC: Ovarian cancer; BTCC: Bladder transitional cell carcinoma; CRC: Colorectal cancer; HR: Hazard ratio; DFS: Disease-free survival; OS: Overall survival; RFS: Recurrence-free survival.

### Correlation of GAS5 expression with clinicopathological features

The main features of the 15 qualified studies on clinicopathological features are summarized in Table 2. Pooled data results (Table 3) showed that low GAS5 expression was significantly correlated with lymph node metastasis (OR = 1.92, 95% CI: 1.44–2.57, *P <* 0.001), distant metastasis (OR = 2.7, 95% CI: 1.05–6.97, *P* = 0.040), poor differentiation (OR = 1.98, 95% CI: 1.40–2.80, *P <* 0.001), larger tumor size (OR = 3.21, 95% CI: 2.08–4.95, *P <* 0.001), and advanced clinical stage (OR = 0.26, 95% CI: 0.18–0.38, *P <* 0.001). There was no correlation between decreased GAS5 expression and either gender or age. Due perhaps to the insufficient data, we failed to detect an association between GAS5 expression and other clinicopathological parameters.

**Table 2:** Main characteristics of the eligible studies that included clinicopathological features

Study	Tumor Type	Sample size	Test Method	Cut-off	Co-variants
Shi 2013	NSCLC	72	qRT-PCR	NA	LNM; gender; differentiation; tumor size
Cao 2014	CC	102	qRT-PCR	median value	LNM; differentiation
Sun 2014	GC	89	qRT-PCR	median value	LNM; DM; differentiation; gender; tumor size; clinical stage
Tu 2014	HCC	71	qRT-PCR	mean value	LNM; gender; tumor size; clinical stage; age
Yin 2014	CRC	66	qRT-PCR	mean value	LNM; DM; gender; age
Chang 2015	HCC	50	qRT-PCR	mean value	Differentiation; gender; tumor size
Dong 2015	NSCLC	72	qRT-PCR	mean ratio	LNM; DM; differentiation; gender; clinical stage; age
Gao 2015	OC	60	qRT-PCR	NA	LNM; differentiation
Hu 2016	HCC	32	qRT-PCR	mean value	Gender; clinical stage
Li 2016	OC	63	qRT-PCR	median ratio	LNM; DM; differentiation; tumor size; clinical stage
Meng 2016	GC	55	qRT-PCR	NA	LNM; differentiation; gender; age; tumor size; clinical stage
Wu 2016	NSCLC	48	qRT-PCR	NA	LNM; differentiation; gender
Xue 2016	PC	118	qRT-PCR	median ratio	Clinical stage
Li 2017	CRC	24	qRT-PCR	NA	LNM; gender; clinical stage
Tan 2017	NSCLC	80	qRT-PCR	Youden index	LNM; gender; age; clinical stage

NSCLC: Non-small cell lung cancer; CC: Cervical cancer; GC: Gastric cancer; HCC: Hepatocellular carcinoma; CRC: Colorectal cancer; OC: Ovarian cancer; PC: Prostate cancer; LNM: Lymph node metastasis; DM: Distant metastasis; NA: not available.

**Table 3:** Meta-analysis results of the correlation of decreased GAS5 expression with clinicopathological parameters

Clinicopathological parameter	Sample size	OR (95% CI)	*P*	Heterogeneity	
				*I*^*2*^	*P*_*h*_
Age (≥ 60 vs. < 60)	344	0.90 (0.58–1.40)	0.645	9.9%	0.350
Gender (Male vs. Female)	659	1.43 (0.98–2.00)	0.059	0.0%	0.917
Clinical stage (I/II vs. III/IV)	604	0.26 (0.18–0.38)	*<* 0.001	0.0%	0.608
Differentiation (Poor vs. Well/Moderate)	602	1.98 (1.40–2.80)	*<* 0.001	48.4%	0.050
Lymph node metastasis (Yes vs. No)	802	1.92 (1.44–2.57)	*<* 0.001	77.6%	*<* 0.001
Distant metastasis (Yes vs. No)	290	2.7 (1.05–6.97)	0.040	2.4%	0.38
Tumor size (≥ 5 cm vs. < 5 cm)	395	3.21 (2.08–4.95)	*<* 0.001	13.9%	0.325

### Diagnostic value of GAS5 expression

**Figure 6:**
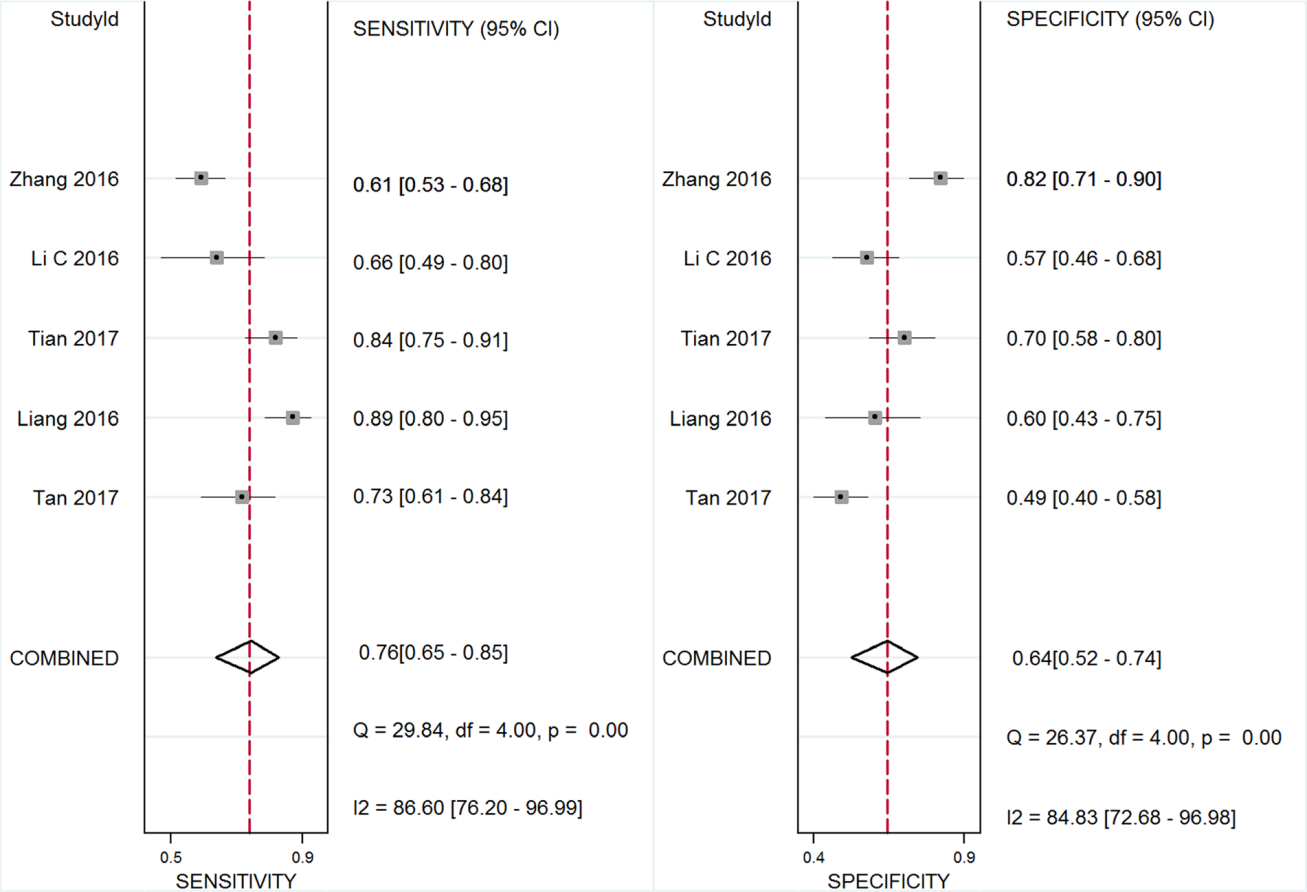
Forest plot of sensitivity and specificity of GAS5 for the diagnosis of cancers

**Figure 7:**
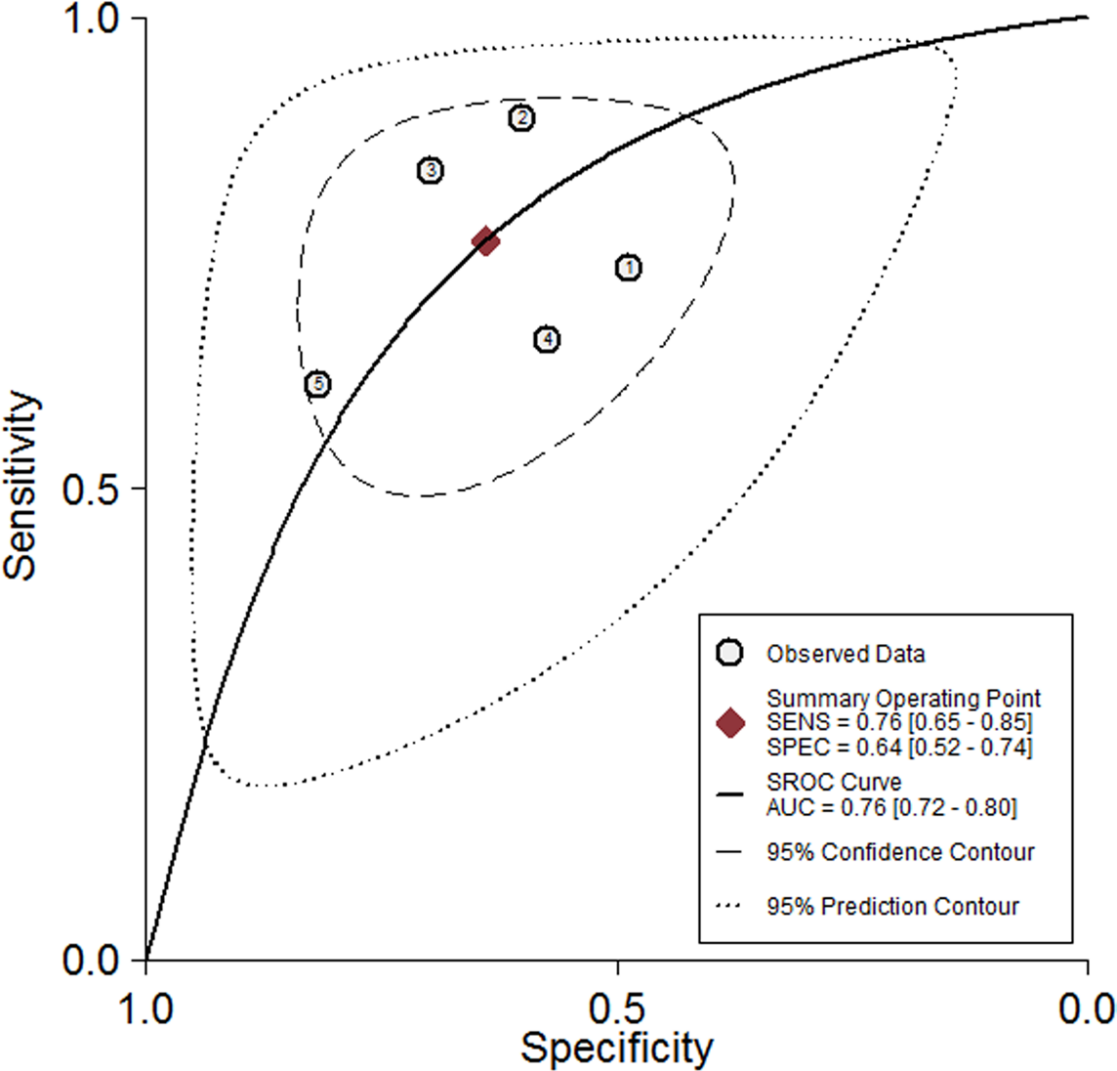
The pooled receiver operating characteristic (SROC) curve based on GAS5

**Figure 8:**
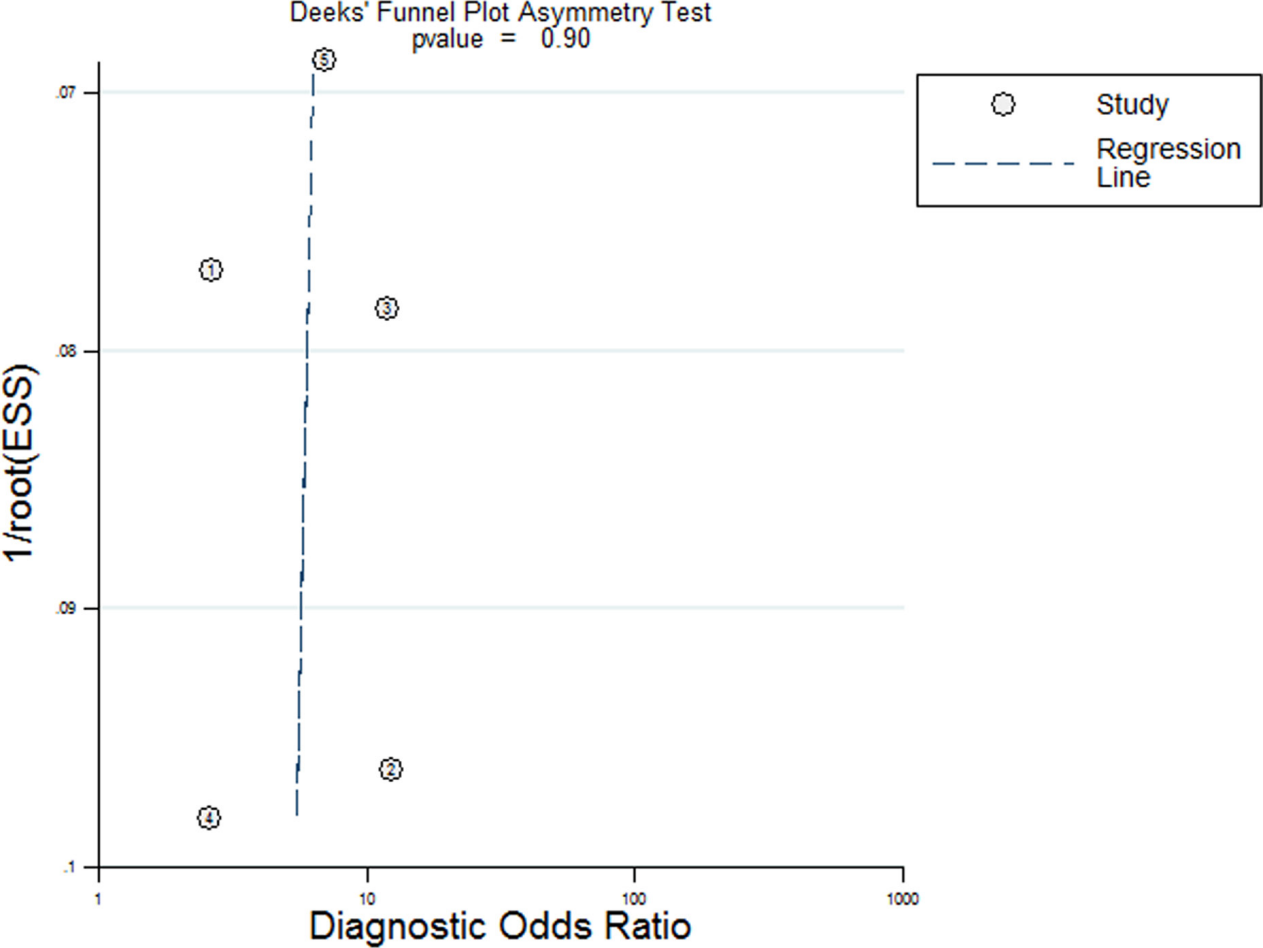
Deeks funnel plot for evaluation publication bias

**Figure 9:**
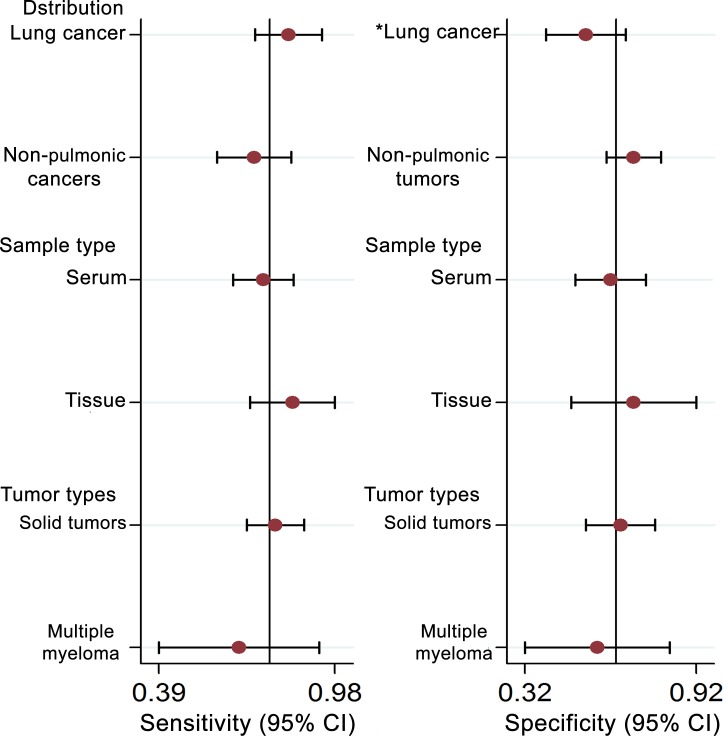
Univariate meta-regression and subgroup analysis for sensitivity and specificity of GAS-5 for the diagnosis of cancers (*P < 0.05)

Five studies, including 477 cancer patients and 366 controls comprising both patients with other diseases and healthy individuals, were used to analyze the diagnostic value of GAS5 expression [13, 23–26]. The main features of the studies are summarized in Table 4. They included data related to four tumor types: NSCLC, HCC, MM, and CRC. The specimens analyzed were serum, in four cases, and tissue in the remaining one. Forest plots depicting sensitivity (SEN) and specificity (SPE) of GAS5 expression for tumor diagnosis are shown in Figure 6. Significant heterogeneity among the studies was observed with respect to both sensitivity and specificity (I^2^ = 86.60% and I^2^ = 84.83%, respectively), hence a random-effects model was applied to summarize the diagnostic parameters. Pooled SEN and SPE values were 0.76 (95%CI, 0.65–0.85) and 0.64 (95%CI, 0.52–0.74), respectively. Pooled positive likelihood ratio (PLR), negative likelihood ratio (NLR), and overall diagnostic odds ratio (DOR) were 2.1 (95%CI: 1.6–2.8), 0.37 (95%CI: 0.25–0.55), and 6 (95 % CI: 3–10), respectively. The area under the summary receiver operating characteristic (SROC) curve (AUC) was 0.76 (95 % CI, 0.72 - 0.80) (Figure 7). These results highlight a potential diagnostic value for lncRNA GAS5. No evidence of publication bias was detected by Deeks’ funnel plot (*P* > 0.05) among the studies (Figure 8). Subsequently, we performed meta-regression and subgroup analysis based on tumor distribution and sample and tumor types (Figure 9). We found that both sample and tumor types were unlikely to affect the overall diagnostic accuracy. However, results suggest that downregulated GAS5 expression may help diagnose lung tumors with higher specificity.

**Table 4:** Summary of GAS5 expression levels as diagnostic cancer biomarker

Study	Tumor type	Sample size		SE (%)	SP (%)	AUC	95%CI	Sample
		**Cases**	**Controls**					
Liang 2016	NSCLC	90	33	82.2	72.7	0.832	0.754–0.893	Serum
Li C 2016	MM	60	60	42	79	0.782	0.700–0.864	Serum
Zhang 2016	HCC	117	129	87.7	48.5	0.734	0.673–0.796	Serum
Tan 2017	NSCLC	111	78	42.31	77.78	0.638	0.515–0.760	Serum
Tian 2017	CRC	99	66	81.9	78.2	0.773	0.484–0.933	Tissue

NSCLC: Non-small cell lung cancer; MM: Multiple myeloma; HCC: Hepatocellular carcinoma; CRC: Colorectal cancer.

## DISCUSSION

Abnormal expression of lncRNAs has been linked to tumorigenesis and tumor progression, and is highly correlated to clinical outcome in several types of cancers [6, 27–29]. GAS5, a prominent non-protein coding RNA gene, is down-regulated in a wide variety of cancer cells and tumor tissues, and has been ascribed tumor suppressor functions [4–6, 10, 15, 21, 30]. GAS5 participates in tumor development by interacting with diverse molecular partners. For instance, GAS5 inhibits the growth of melanoma cells by promoting miR-137 transcription [30], and its upregulation suppresses cell proliferation and promotes apoptosis of human glioma cells by inhibiting miR-222 expression [31]. In gastric cancer cells, GAS5 was reported to inhibit proliferation by upregulation of p21 and suppression of CDK6 [32]. In addition, downregulation of GAS5 has been associated with chemoresistance in some malignant neoplasms, by affecting the sensitivity to adriamycin in gastric cancer [33], trastuzumab in breast cancer [34], mTOR inhibition in prostate cancer [35], and EGFR tyrosine kinase inhibition in wide-type EGFR NSCLC [20]. This evidence indicates that GAS5 can act as a common molecular marker for monitoring the effect of chemotherapy in several tumor types.

To analyze the results of previous studies evaluating the relationship of GAS5 expression with cancer diagnosis, clinicopathological parameters, and prognosis, we implemented this comprehensive meta-analysis. A total of 21 eligible studies, addressing 8 common cancer types, met the selection requirements for inclusion. Survival data comprised OS, DFS, and RFS. The results indicated that decreased GAS5 expression is significantly correlated with unfavorable clinical prognosis in patients with various tumor types. First, pooled results demonstrated that decreased GAS5 expression was associated with a shorter OS time, suggesting that GAS5 may serve as a potential independent predictive biomarker for OS in cancer patients. Second, reduced GAS5 levels were significantly associated with unfavorable DFS or RFS. Third, aggregated results indicated that decreased GAS5 expression was positively correlated with poor tumor differentiation, larger tumor size, and advanced clinical stage. In addition, cancer patients with low GAS5 expression in tumor tissues were more prone to develop LNM and DM. These results indicate that GAS5 could be a useful biomarker for LNM and DM at early tumor stages. However, due to the paucity of studies available in this regard, this conclusion should be further verified.

In diagnostic analyses of tumor biomarkers, a PLR greater than 10 and an NLR lower than 0.1 generally denote satisfactory diagnostic performance [36]. The aggregated PLR and NLR obtained in our analysis (2.1 and 0.37) suggest that GAS5 expression has at best moderate accuracy in cancer diagnosis. Due to the significant heterogeneity observed during pooled analysis of sensitivity and specificity, we executed subgroup analyses on tumor distribution, and sample and tumor types. The results showed that sample source and tumor types were unlikely to affect the diagnostic accuracy of GAS5. However, we found that GAS5 expression may be a more specific diagnostic biomarker of lung cancer, compared with other neoplasms. The AUC of 0.76 calculated for GAS5 in tumor diagnosis also suggests moderate diagnostic value. According to published research, improved diagnostic efficacy could be achieved by combining expression data for diverse lncRNAs. For instance, Li et al. [25] found that GAS5 and CRNDE-h combined have a higher positive diagnostic rate for patients with multiple myeloma than GAS5 or CRNDE-h alone. Also, a higher pooled AUC was computed for the combination of lncRNA-LET, PANDAR, PVT1, linc00963, and PTENP1, compared to each of those lncRNAs alone [37]. Strategies like these may prove to be a more successful approach to determine both common and disease-specific molecular markers for tumor diagnosis.

Our meta-analysis presents some limitations that are worth examining. First, the number of original research studies and the total sample size included in our meta-analysis were relatively small, so more qualified studies are needed to perform a more thorough study. Second, since all the included cancer patients were Asians from China, our meta-analysis lacks clinical data from other races, needed to generalize our conclusions. Thirdly, some HRs and their corresponding 95%CIs were extracted from Kaplan-Meier curves, and may be less reliable than those directly obtained from survival data. Finally, given the significant heterogeneity detected in the analysis of the diagnostic value of GAS5 expression, and the limited diagnostic capability hence derived, further basic and analytical research is warranted to confirm and validate the present results.

In conclusion, our meta-analysis indicates that decreased GAS5 expression is positively correlated with poor tumor differentiation, larger tumor size, and advanced clinical stage. Furthermore, our results suggest that low GAS5 expression is a risk factor for LNM and DM in diverse cancers. Well designed, larger-size, and higher-quality basic studies need to be conducted to validate the clinical value of GAS5 as a diagnostic and prognostic cancer marker.

## MATERIALS AND METHODS

### Literature search strategy

A literature search (up to March 20, 2017) was conducted on the online electronic databases Embase, PubMed, Web of Science and CNKI. Search keywords or their combinations were: “GAS-5 OR GAS 5 OR growth arrest-specific 5” AND “cancer OR carcinoma OR tumor OR tumour OR neoplasm OR gliomas OR angiosarcoma OR lymphoma OR melanoma OR leukemia”. Only articles in English or Chinese were included in this study.

### Study selection criteria

The criteria for study inclusion in this meta-analysis were as follows: (1) studies evaluating the association of GAS5 with clinicopathological features, with expression levels of GAS5 divided into two groups: high or low; or (2) studies that detected GAS5 levels in serum or tissue and contained adequate data to construct a two by two diagnostic table; or (3) studies that provided sufficient data for computation of odds ratio (OR) or hazard ratio (HR) with 95% confidence interval (CI), and Kaplan-Meier curves or, if unavailable, pertinent data obtained by contacting the corresponding authors.

The study exclusion criteria included the following: (1) duplicate articles; (2) case reports, letters, expert opinions, commentaries, editorials, and reviews; (3) studies without available data; (4) sample cases fewer than 30; (5) non-human research.

### Data extraction

Two investigators (Wei Li and Na Li) independently extracted and reviewed the data from each eligible study. Data collected included first author’s name, publication date, study location, tumor type, tumor stage, GAS5 expression detection method, assessment criteria for GAS5 expression, sample size, total patient number, number of patients in the high and low GAS5 expression groups, number of patients with lymph node metastasis (LNM) and distant metastasis (DM) in each group, survival data analysis, follow-up period, sample sizes and statistical results (sensitivity, specificity, AUC) for diagnostic/prognostic analyses and two by two table construction, and OR/HR and corresponding 95% CI. If the articles did not show survival data, a request was made to the corresponding authors, or data were extracted from survival plots, with HRs estimated by using Engauge Digitizer v.4.1 software as previously described [38, 39].

### Statistical methods

The STATA 12.0 software (Stata, College Station, Texas) was used to perform all statistical analyses. GAS5 expression was categorized into a high expression and a low expression group according to the original published articles. Heterogeneity among the included studies was determined using the I-squared statistic, with I^2^ values greater than 50% suggesting that substantial heterogeneity was present. A fixed effects model was used to analyze the pooled results when the included studies showed moderate heterogeneity (I^2^ < 50 %). Otherwise, a random effects model was employed (I^2^ > 50%). Sensibility analysis was conducted to evaluate the robustness of the overall results. Begg’s funnel plot was used to assess for potential publication bias. For the prognosis meta-analysis, the ln(HR) and standard error were used to integrate survival results. Also, sensitivity, specificity, PLR, NLR, DOR, SROC curve, and AUC tests and measures were applied to assess the diagnostic accuracy of GAS5. Meta-regression and subgroup analysis were utilized to investigate the origin of heterogeneity. All the *P*-values were determined by a 2-tailed test and *P* < 0.05 was regarded as statistically significant.
